# Evidence of prescription of antidepressants for non-psychiatric conditions in primary care: an analysis of guidelines and systematic reviews

**DOI:** 10.1186/1471-2296-14-55

**Published:** 2013-05-04

**Authors:** Alain Mercier, Isabelle Auger-Aubin, Jean-Pierre Lebeau, Matthieu Schuers, Pascal Boulet, Jean-Loup Hermil, Paul Van Royen, Lieve Peremans

**Affiliations:** 1 Department of General Practice, Rouen University and CIC Inserm 0204, University of Rouen, Rouen, France; 2 Department of General Practice, Denis Diderot Paris 7 University, Paris, France; 3 Department of General Practice, Tours University, Tours, France; 4 Department of Primary and Interdisciplinary Care, Faculty of Medicine and Health Science, University of Antwerp, Antwerp, Belgium; 5 Department of Public Health, Vrije Universiteit Brussel, Brussels, Belgium

**Keywords:** Antidepressants, Literature review, Therapeutic use, Family practice

## Abstract

**Background:**

Antidepressants (ADs) are commonly prescribed in primary care and are mostly indicated for depression. According to the literature, they are now more frequently prescribed for health conditions other than psychiatric ones. Due to their many indications in a wide range of medical fields, assessing the appropriateness of AD prescription seems to be a challenge for GPs. The aim of this study was to review evidence from guidelines for antidepressant prescription for non-psychiatric conditions in Primary Care (PC) settings.

**Methods:**

Data were retrieved from French, English and US guideline databases. Guidelines or reviews were eligible if keywords regarding 44 non-psychiatric conditions related to GPs’ prescription of ADs were encountered. After excluding psychiatric and non-primary care conditions, the guidelines were checked for keywords related to AD use. The latest updated version of the guidelines was kept. Recent data was searched in the Cochrane Database of Systematic Reviews and in PubMed for updated reviews and randomized control trials (RCTs).

**Results:**

Seventy-eight documents were retrieved and were used to assess the level of evidence of a potential benefit to prescribing an AD. For 15 conditions, there was a consensus that prescribing an AD was beneficial. For 5 others, ADs were seen as potentially beneficial. No proof of benefit was found for 15 conditions and proof of no benefit was found for the last 9. There were higher levels of evidence for pain conditions, (neuropathic pain, diabetic painful neuropathy, central neuropathic pain, migraine, tension-type headaches, and fibromyalgia) incontinence and irritable bowel syndrome. There were difficulties in summarizing the data, due to a lack of information on the level of evidence, and due to variations in efficacy between and among the various classes of ADs.

**Conclusions:**

Prescription of ADs was found to be beneficial for many non-psychiatric health conditions regularly encountered in PC settings. On the whole, the guidelines were heterogeneous, seemingly due to a lack of trials assessing the role of ADs in treatment strategies.

## Background

Antidepressants (ADs) are commonly prescribed in primary care (PC). Among the general population, the 12 month prevalence of ADs consumption ranges from 6% to nearly 10% [1,2]. The main indications for ADs are major depressive episodes and anxiety. Over the past 20 years, the use of ADs has grown extensively. Most studies have shown a high level of consumption in all industrialised countries. In France, between 1980 and 2008, AD sales increased sevenfold, from €84 million to €525 million per year [3]. Selective Serotonin Reuptake Inhibitors (SSRIs) and Selective Norepinephrine Reuptake Inhibitors (SNRIs) accounted for about 80% of sales. Data in various industrialised countries showed similar results [4-7]. In the United States, ADs are the third most commonly prescribed medication [8,9]. This growing prescription rate is a source of concern for healthcare providers and healthcare economists alike [8,10]. The explanations for this high prescription rate remain unclear, with little consensus, and the reasons behind the phenomenon remain largely unknown [11,12]. According to the literature, there are two main causes for this situation. The first is over-prescription for psychiatric conditions. Evidence does not show ADs to be highly clinically effective in treating moderate depression, which is frequently encountered in PC settings, although several recent studies have found that ADs could possibly be beneficial in treating milder episodes [13,14]. ADs are sometimes discontinued too early or prescribed too long [15,16]. Assessing the potential benefit of AD prescription seems to be a challenge, as the variations in measurement specifications in the studies impact the conclusions that are drawn about treatment of depression [17]. The second reason is the prescription of ADs for “non-psychiatric conditions”. Growing evidence points to ADs being frequently prescribed for conditions or health problems outside the field of psychiatry [18]. Some observational data suggest that this proportion varies between 25% and 60% [19,20]. Exploratory research has confirmed that GPs prescribe ADs for many non-psychiatric conditions and off-label uses. In many fields, GPs used their feelings and feelings on the products’ efficacy rather than scientific evidence to prescribe. [21]. The aim of this study was to review the level of evidence for the prescription for ADs in non-psychiatric PC conditions in order to help GPs in their daily practice.

## Methods

**Figure 1:**
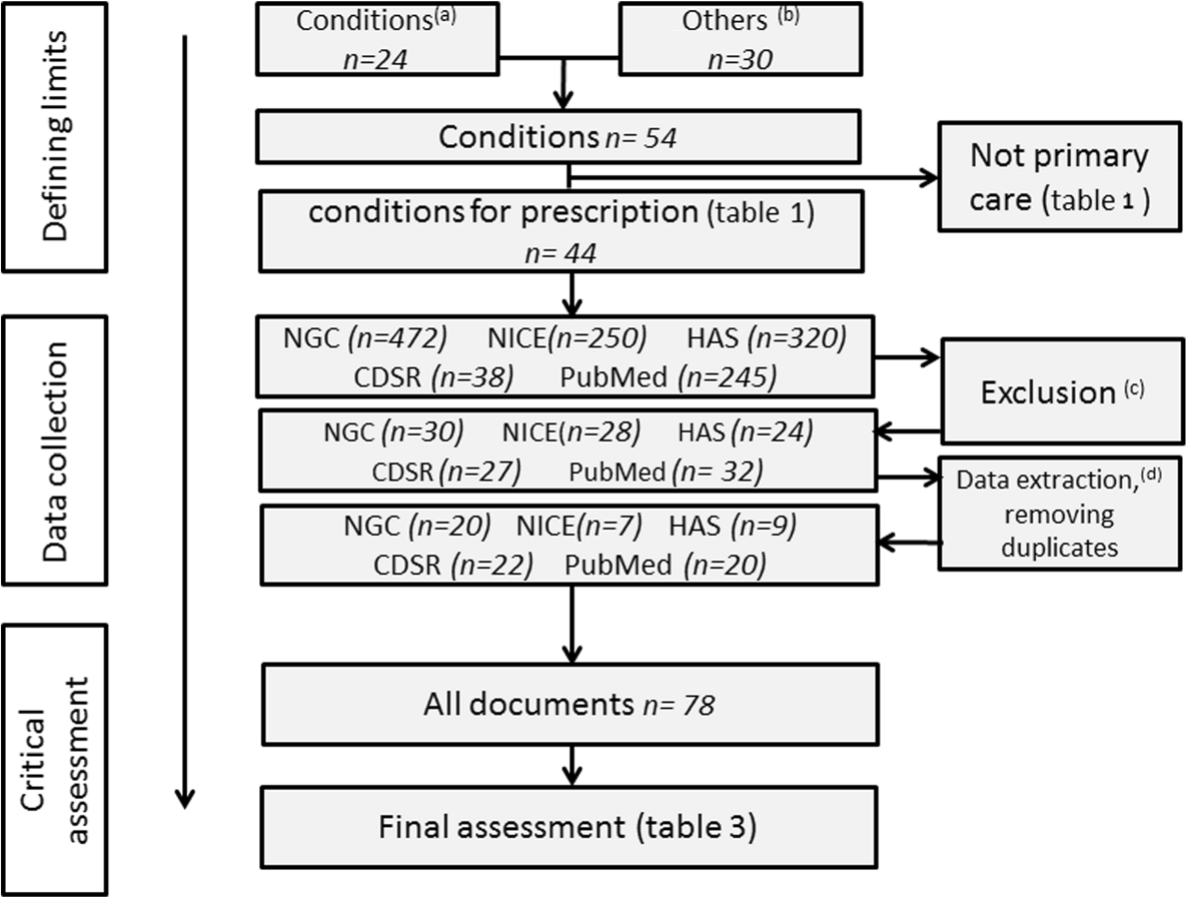
**Methods and overall process. (a) **Conditions mentioned by the GPs ^ref^. **(b) **Conditions collected via Pubmed searches. **(c) **Guidelines before 1997, paediatrics, nursing practices, patient information or education, medical records, continuous medical education for care providers, medical imaging, biology and surgical techniques. **(d) **Selection of the latest guidelines containing the key words (antidepressant”, “Tricyclic agents”, “TCA”, “SNRI”, “serotonin”, “SSRI”, “tricyclic”, “imipramine”, “monoamine”, “duloxetine”, “venlafaxine).

The overall review process is summarised in Figure 1.

### Defining the limits of the searches

To retrieve the level of evidence for the prescription of ADs in non-psychiatric PC settings, an initial search was performed using the MeSH terms for the 24 medical conditions collected in our previous qualitative study and the terms “antidepressants” and “therapeutic use” in PubMed [21]. During this process, 30 additional conditions possibly leading to AD prescription were found resulting in a list of 54 conditions or medical circumstances for which ADs could possibly be prescribed. Ten conditions - psychiatric disorders, non-primary care and non-adult conditions - were then excluded: five purely psychiatric disorders, narcolepsy and myotonia because of their low prevalence in primary care, nocturnal enuresis as a paediatric condition and amphetamine withdrawal and cocain dependence being conditions to be managed in multidisciplinary and specialised environment. This process resulted in a list of 44 non-psychiatric disorders manageable in PC and potentially leading to AD prescription in PC (Table 1). This list was used for the data collection search strategy.

**Table 1:** Conditions included and excluded in the searches


**Conditions included: primary care, non-psychiatric***n = 44*	**Pain***: n = 14*
	Neuropathic pain, diabetic painful neuropathy, HIV-related neuropathy, trigeminal neuralgia, post herpetic neuralgia, phantom limb pain, central neuropathic pain, burning mouth syndrome, migraine, tension-type headaches (with or without drug abuse), fibromyalgia, non-specific low back pain, sciatica
	**Other neurological conditions or symptoms:***n = 9*
	Dementia (agitation), Parkinson’s disease (depression / agitation), emotionalism after stroke, prevention of depression after stroke, motor recovery after ischemic stroke, sleep disorders, restless legs syndrome, sialorrhea, tinnitus
	**Urological conditions:***n = 5*
	Urinary incontinence, overactive bladder syndrome, urinary stress incontinence, erectile dysfunction, premature ejaculation
	**Dependence:***n = 2*
	Smoking cessation, alcoholism
	**General or non-specific conditions, general symptoms:***n = 10*
	“Chronic fatigue Syndrome or Asthenia or Fatigue”, cancer-related fatigue, depression in physically ill people, musculoskeletal symptoms, unexplained complaints, somatoform disorders, treatment refusal, patient compliance, weight loss in adults with type 2 diabetes mellitus, pruritus
	**Gynaecological conditions:***n = 3*
	Premenstrual syndrome, hot flashes/drug therapy, menopause
	**Gastroenterological conditions:***n = 1*
	Functional colonic diseases: irritable bowel syndrome
**Conditions excluded: **^1^*n = 10*	Narcolepsy, anorexia nervosa, isolated depression, dysthymic disorder, all isolated anxiety conditions, bulimia nervosa, amphetamine withdrawal, nocturnal enuresis, myotonia, cocaine dependence

### Data collection: search strategy

The terms matching with the 44 conditions and medical circumstances for possible AD prescription were listed in French and English. The MeSH validated translation and synonyms were searched for in the terminology section of the catalogue and index of the French-language Health Resources Website (CISMeF). When there was no MeSH term available for the medical condition or circumstance, the researchers used a broader MeSH term, on a higher level of hierarchy, including the description (e.g., cancer AND antidepressants for “cancer related fatigue, musculoskeletal diseases AND antidepressants for musculoskeletal symptoms). Using this list, searches were performed to find the available guidelines (GL) in the databases of the following government agencies: the French “Haute Autorité de Santé” (HAS), the English National Institute for Clinical Excellence (NICE) and the National Guideline Clearinghouse (NGC), the last one including most international guidelines. The searches were performed from March 2011 to August 2011. The search covered all of the documents made available by the three agencies.

### Data collection: additional searches for updated data

During the same period, we performed simultaneously, a search in the Cochrane database of systematic reviews (CDSR) and in Medline on the same basis, using the same criteria and process. For the Medline search, the following limits were used: “clinical trial, meta-analysis, clinical guidelines, randomized controlled trial, and adults, published after 2004”. The aim was to find scientific data published after the launch of the guidelines. Those studies might change the evidence and possibly lead to a change in prescription attitudes. All detailed searches are available in the full version of all guidelines. Individual studies (RCTs in Pubmed) included in this review did not constitute the bulk of the results.

### Data extraction

All guidelines before 1997 were excluded, as were those regarding paediatrics, nursing practices, patient information or education, medical records, continuing medical education for care providers, medical imaging, biology and surgical techniques. Full text of guidelines have been downloaded from the agencies databases. All non-psychiatric conditions were included and all standalone psychiatric conditions were excluded. Remaining documents on anxiety, depression and side effects of ADs were also excluded after reading. The documents were independently read and assessed by two researchers (AM & IAA) and disagreements were resolved by discussion. Duplicates were removed. The documents in the databases were crosschecked using a search with the generic term “antidepressants” in order to retrieve any documents that may have been missed by previous searches. In the full text documents, the terms “antidepressant”, “Tricyclic agents”, “TCA”, “SNRI”, “serotonin”, “SSRI”, “tricyclic”, “imipramine”, “monoamine”, “duloxetine”, “venlafaxine” and their French translations were searched for. All guidelines containing the keywords were selected. In order to find the latest evidence, the most recent documents available on each topic in each guideline database were retained when available, and the most up-to-date version of the selected guidelines was kept. The overall process ended with a list of 78 documents.

### Critical assessment

The final stage of the study aimed to retrieve guidelines on recommended therapeutic use of ADs from these documents. Sections of specific interest were extracted and analysed. Information on pharmaceutical classes, drugs, recommended therapeutic use and level of evidence was collected when available. Agreements and discordances were noted, and inconsistencies between the various guidelines were highlighted. Recommendations from the guidelines were graded according to the EFNS scheme [22] (Additional file 1).

## Results

We collected 1,325 documents on 44 conditions. After exclusion, 141 remained. After applying our criteria for data extraction, 78 documents were retrieved, including 36 GLs, 38 reviews or systematic reviews (some followed by a meta-analysis) and 4 RCTs. The guidelines enabled us to describe the evidence for AD prescription for 27 conditions. For 14 conditions, searches in CDSR and PubMed retrieved updated information. For 3 conditions (somatoform disorders, treatment refusal, and patient compliance), no data were found. Among the 44 conditions, ADs were found to be potentially beneficial with a high level of evidence in treating 15 conditions and potentially beneficial with a lower level of evidence in treating 5 others. No proof of benefit was found for 15 conditions and proof of no benefit for 9. All results are detailed in Table 2.

**Table 2:** Assessment of AD usefulness


	-Neuropathic pain (neuralgia and painful polyneuropathy), diabetic painful neuropathy, central neuropathic pain, migraine, tension-type headaches, fibromyalgia
**Useful **^**(1)**^	-Urinary stress incontinence, premature ejaculation
*n = 15*	-Prevention of depression after stroke, emotionalism after stroke -Smoking cessation
	-Premenstrual syndrome, hot flashes/drug therapy, hot flashes during menopause
	-Irritable bowel syndrome
**Possibly useful **^**(2)**^	-Post herpetic neuralgia, trigeminal neuralgia
*n = 5*	-Agitation in dementia, motor recovery after ischemic stroke
	-Overactive bladder syndrome
**No proof of benefit **^**(3)**^	- Tension-type headaches with drug abuse, sciatica, Parkinson’s disease, sleep disorders
*n = 15*	- pruritus
	- Asthenia- fatigue-chronic fatigue syndrome, cancer-related fatigue, depression in physically ill people
	- Unexplained complaints, somatoform disorders, treatment refusal, patient compliance, weight loss in adults with type 2 diabetes
**Proof of no benefit **^**(4)**^	-HIV related neuropathy, phantom limb pain, burning mouth syndrome, non-specific low back pain, restless legs syndrome
*n = 9*	-Other urinary incontinence conditions, erectile dysfunction -Alcoholism /
	alcohol misuse
	-Musculoskeletal symptoms ^(5)^

(1) Recommended with evidence level mentioned or recent meta-analysis. (2) Mentioned without level of evidence, or second line treatment, or only RCTs. (3) Not enough or no data, apart from psychiatric condition. (4) No benefit mentioned in RCTs or reviews. (5) See fibromyalgia in Table 3.

### Pain conditions

All results for pain conditions are described in Table 3.

**Table 3:** **ADs in pain conditions **^1^

***Condition***	***AD treatment, role in strategy, rating of the recommendation***	***Comments and role of other treatments***
**Neuralgia and painful polyneuropathy**[22-26]	*ADs recommended:*	-Similar statements between guidelines:
	**-***TCAs: *Amitriptyline first line (25–150 mg/day, Level A) Nortriptyline (alternative option) [22-26] NNT of 3.6 (95% CI: 3-4.5).	-Strong consensus for TCAs and venlafaxine.
	*-SNRIs: *Venlafaxine, fist line treatment (Level A) NNT of 3.1 Duloxetine: option	-Gabapentin, Pregabalin: also recommended as first-line treatments. TCAs are equally effective compared to non-AD drugs gabapentin (1200–3600 mg/day) and pregabalin (150–600 mg/day)
	*SSRI: not recommended*	
**Painful Diabetic Neuropathy (PDN)**[25-29]	*ADs recommended:*	
	-Duloxetine 60 mg and 120 mg daily, first-line, (Level A) The NNT for effectiveness was 1.3 (95% CI: 1.2- 1.5). This AD has on-label use for this condition [25,26,28]	Duloxetine: conflicting evidence between guidelines, just cited as a therapy for NP in the EFNS GL[24], and only for PDN in the Cochrane Review
	-Venlafaxine 150–225 mg/day; first line (no level mentioned)TCA : If other ADs contraindicated, Amitriptyline is an option	Venlafaxine might be added to gabapentin for a better response (Level C).
	*SSRIs: not recommended*	
**HIV-related neuropathies**[27]	*No AD treatment recommended*	-Evidence not to prescribe any AD
		- Recommended non-AD treatments: -lamotrigine (Level B), smoking cannabis (Level A), capsaicin patches (Level A)
**Phantom limb pain**[30]	*No AD treatment recommended*	Amitriptyline was not different from placebo
**Trigeminal neuralgia**[25,26]	*-ADs are not first-line treatment*	-Similar statements but lack of comparative trials to assess a precise role for TCAs and venlafaxine.
	*-ADs recommended: *TCA or venlafaxine are alternative treatments	First-line: carbamazepine (Level A) and oxcarbazepine (Level B)
**Postherpetic neuralgia**[25,26]	-*ADs are not first-line treatments*	-Similar statements but lack of comparative trials to assess a precise role for TCAs and venlafaxine
	*- ADs recommended: *TCA or venlafaxine are alternative treatments	-First-line: gabapentin / pregabalin (Level A)
**Central pain **^2^[24]	*-ADs are not first-line treatment*	-Similar statements between guidelines
	*- ADs recommended: *TCAs: Amitriptyline second-line (Level B)- SNRIs: Duloxetine and venlafaxine second choice (Level B)	-Pregabalin: first-line (level A)
**Migraine and tension type headaches**[31-34]	*ADs recommended:*	-Similar statements for TCAs, and SSRIs. Disagreement for the usefulness of venlafaxine
	-TCA: Amitryptiline 25-150 mg per day, (Level A).-Venlafaxine 75-150 mg was presented as an effective alternative to tricyclic antidepressants (Level B)	-TCA: In cases of TTH with associated drug abuse, the role of this treatment was only mentioned, with no rating, by the French HAS.
	*SSRIs: not recommended*	
**Sciatica, non-specific low back pain**[35-37]	*No AD treatments recommended*	Only to be prescribed as an option in the event of associated depression (NICE)
		Very weak evidence for TCAs observed by the French HAS (level C)
**Fibromyalgia**^3^[28,38]	*ADs recommended:*	Alternative pharmacological options: Gabapentin, tramadol
	SNRIs: Milnacipran 12.5 mg once daily, target dose of 50-100 mg two times per day	
	-Duloxetine: 60 mg twice daily, -Venlafaxine could be prescribed -TCAs showed evidence	
	*SSRIs: not recommended*	
**Burning mouth syndrome**[39]	*No AD treatments recommended*	-Two RCTs showed no antidepressant effects

(1) Neuropathic pain is related to different treatment strategies and different conditions detailed in this table.

(2) Diffuse pain, refractory or recurrent pain, central pain, pain connected with multiple sclerosis, dysesthesia after stroke or paraplegia.

(3) See also comments in plain text: *General or non-specific conditions and general symptoms.*

### Neurological conditions

The guidelines, reviews and RCTs found prescription of sertraline, citalopram and trazodone to be potentially beneficial in treating behavioural perturbations, mood disorders and agitation in patients with dementia, though no level of evidence was available [40,41]. Potential side effects and difficulty in managing the prescription were emphasised [42]. ADs were found to have no specific effects in treating Parkinson’s disease, apart from those in treating its associated psychiatric indications [43].

Concerning stroke, three kinds of problems were assessed: treatment of emotionalism, prevention of depression and the benefit of prescribing an AD in the acute stage to facilitate recovery of motor skills. ADs were recommended for emotional instability (Level B) [44,45]. They reduced the frequency and severity of crying and laughing episodes. The effect did not seem specific to one drug or class of drugs. Early prescription prevented depression, but no improvement in its severity was found when depression was actually occurring [46]. Early prescription of fluoxetine with physiotherapy found that patients with ischemic stroke and moderate to severe motor deficit could enhance motor recovery after 3 months [47].

Antidepressants were found to provide no benefit in treating isolated sleeping disorders and primary insomnia even though they were found to be potentially beneficial in the event of psychiatric comorbidity [48].

Antidepressants were not recommended for use in cases of restless legs syndrome, which was in fact presented as a side effect of ADs [49].

As well, there was no evidence of a benefit in prescribing ADs for cases of sialorrhea related to neurological conditions (Amyotrophic lateral sclerosis (ALS), Parkinson’s disease), although prescription was sometimes recommended [50]. Antidepressants were found to have no proof of benefit for cases of tinnitus [51].

### Urological and gynaecological conditions

Duloxetine was found to have potential benefits as a second-line (Level C) treatment for patients with stress urinary incontinence. Duloxetine significantly improved quality of life but TCAs did not. Anticholinergic agents, such as TCAs, were found to have potential benefits for patients with overactive bladder syndrome [52-54]. Proof of benefit was observed for incontinence caused by other urological conditions. Antidepressants were cited as a potential source of side effects for these conditions [55].

One Cochrane review attested that all SSRIs were highly effective in reducing symptoms related to severe premenstrual syndrome, (also called pre-menstrual dysphoric disorder or luteal phase dysphoric disorder) (SMD -0.53, 95% CI: 0.68 to -0.39; P < 0.00001) with no level of evidence available [56]. Another Cochrane review reported that SSRIs and SNRIs had a mild to moderate effect in reducing hot flashes during menopause in women with a history of breast cancer, as well as in men with a history of prostate cancer (Level B) [57].

SSRIs were not recommended for erectile dysfunction. They were, however, recommended as a first-line (Level A) treatment for premature ejaculation [58,59].

### Dependence

Nortriptyline was shown to be effective for tobacco withdrawal (OR 2.79 (95% CI: 1.70-4.59) whereas moclobemide, venlafaxine and SSRIs did not show any effectiveness (no level of evidence available) [60]. All guidelines and reviews agreed that ADs were not indicated for alcohol misuse or dependence [61]. It is clearly stated that depression is a direct consequence of alcohol abuse, and that AD prescription is useless if the patient does not stop drinking.

### General or non-specific conditions and general symptoms

The term “fatigue” pooled together a wide variety of health problems. No data was retrieved for isolated fatigue. In the NICE guideline, ADs were not found to be beneficial in treating chronic fatigue syndrome. ADs were considered as useless for cancer-related fatigue but beneficial in treating depression related to purely physical conditions, with no effect on the physical conditions themselves [62].

Prescribing an AD provided no benefit for musculoskeletal symptoms, except for fibromyalgia, which is assessed in the “pain conditions” section of the results (see also Table 3) [63]. No data was retrieved regarding prescription for unexplained complaints, somatoform disorders, treatment refusal, chronic pruritus, helping type 2 diabetes patients to lose weight, and improving medical adherence or patient compliance.

### Gastroenterological conditions

According to the CDSR, TCAs could be used as a second-line treatment and SSRIs as a third-line treatment, for irritable bowel syndrome (IBS) (no level of evidence available) [64,65].

## Discussion

### Summary of the main results

Antidepressants were found to be potentially useful with a high level of evidence for 15 conditions and potentially useful with a lower level of evidence for 5 others. Prescribing an AD was found to be potentially beneficial for patients with many pain conditions as well as urological, gastroenterological and gynaecological conditions. Nevertheless, while prescribing, the GP has to refer to on-label use for his own country, because unfortunately, proof of benefit is not related to “on label use”. The evidence reviewed provided insufficient support for prescribing ADs for 24 other conditions. Restless legs syndrome, non-specific urinary incontinence and erectile dysfunction exposed in the “proof of no benefit” section were side effects of ADs. For rheumatologic conditions, (musculoskeletal symptoms and non-specific low back pain) the “proof of no benefit” should be interpreted with caution, due to the difficulty in assessing these patients in a primary care setting. Pain symptoms or somatoform complaints may be similar to those encountered in fibromyalgia, a clinical syndrome that lacks a clear definition and is still a subject of debate [66]. For the other 15 conditions listed in the “No proof of benefit” section, this should be understood as “having no specific effect on the non-psychiatric condition mentioned”. For example, no guidelines found ADs to have a specific effect in treating Parkinson’s disease comparable to their effect in treating dementia-related agitation. Nevertheless, ADs should be prescribed in the event of a major depressive episode related to Parkinson. The same can be said for treating depression in physically ill people or those with cancer [62]. Thus, an AD considered as having no specific benefit for a given condition may prove beneficial for certain specific PC patients. As a result, it is difficult for prescribing physicians to assess the potential benefit of using them in clinical practice in PC settings where several conditions are involved.

### Strengths and limitations of the study

The first strength of the study was the meticulous methodology and careful step-by-step process used to extract the data. The analysis was not started from a theoretical framework, but from real daily practice in primary care, based on the qualitative material of our previous research [21]. No previous study has assessed in that way the appropriateness of AD prescriptions for non-psychiatric conditions. This review gives a new insight on studies emphasizing misuse as an explanation for the increasing rate of AD prescription [67-70]. A second strength was that it provided all GPs a clear overview of information on ADs not limited to the information and guidelines available in their own country. Finally, a third strength of the study was that it updated the guidelines with recent reviews, in order not to miss relevant information. This study, being an analysis of guidelines and systematic reviews did not focus on a single condition, population or intervention, but on a family of drug (ADs) and their indications. Thus, the nature of this analysis made it impossible to perform a systematic review fulfilling the “Prisma” statements. Another limitation of this study was the consistency of the available data. First of all, levels of evidence were not always available. When available, levels of evidence were not always consistent between the different guidelines, as was the case with diabetic painful neuropathy. Additionally, either a given AD, only certain ADs or an entire class of ADs could be considered as having a potential benefit in treating a specific condition. For example, among the SSRIs, only citalopram and sertraline were assessed in treating dementia-related agitation. Also, the optimum dosage to be used in treating a condition was not always determined, as in the case of using amitryptiline to treat migraine or neuropathic pain. Finally a limitation of our study could be that some guidelines in other languages than French and English were not selected in the search. However we used the big databases such as Medline and NGC, which already include guidelines, sometimes translated in English, from many guideline developers worldwide.

Performing a strict assessment and comparison of several conditions appeared arduous: although the subjects of the recommendations were similar, the guidelines did not refer to strictly comparable conditions. For example difficulties were encountered with fibromyalgia, chronic fatigue syndrome and rheumatologic pain, which were difficult to distinguish from one another. This lack of precision can lead to difficulties in assessing and summarizing information, and consequently in correctly understanding the potential benefit of ADs for patients. As well, the randomised trials were designed for well-defined conditions and homogeneous populations, which is rarely the case for PC patients. The inclusion criteria for the studies often involved secondary care and did not reflect primary care situations.

The limitations of available data and the wide variety of conditions made it difficult to formulate clear recommendations. In any case, the results of the study provide new insight to enable clinicians to prescribe ADs with more accuracy, and could potentially serve as a point of reference for health policy organizations in specifying prescription rules.

### Consistency of GPs’ prescriptions with the evidence

Comparing the 24 non-psychiatric health conditions for which the GPs claimed to prescribe ADs [21] with the results of this review study, 12 conditions were found to be based on evidence. Of the 12 other conditions for which GPs claimed a prescription of an AD, there was no scientific evidence. In our qualitative study, the GPs correctly assessed the benefit of ADs in treating pain conditions [21]. This seems contradictory with observational studies finding that pain management could be improved in PC settings [71]. However GPs’ proper assessment of the benefit of ADs, depends on his ability to make a fine distinction between “the scientifically proven effect of an AD for a certain condition” and “the potential benefit of an AD at the right dose for a specific patient presenting with several psychiatric or non-psychiatric conditions”. Besides the GP’s decision is based not only on biomedical observations, but also follows a bio-psychosocial model in which patient-centeredness and the environment are taken into account [67]. The major benefit of this study is to understand better and justify GPs’ AD prescription behaviour.

### Implications for future research

On the whole, the guideline agencies often stressed a lack of head-to-head comparative trials assessing the clinical effectiveness of the drugs, which would allow physicians to prioritise the choice of therapy for a given condition. This is impossible without any involvement from healthcare institutions. Another challenge is to better understand the way physicians prescribe ADs in actual PC settings. We currently know that GPs’ decisions are shaped by a combination of diseases and psychosocial influences. The potential benefit of a prescription for a patient cannot be assessed without incorporating all of the dimensions of actual prescription. This requires a strict comparison that directly assesses the prescriptions, indications and patients’ environment, based on observational cohort studies to collect data on medical conditions, psychosocial environment and prescriptions in PC settings.

## Conclusions

Prescription of ADs was found to be useful for many non-psychiatric health conditions commonly encountered in primary care. Evidence against prescribing ADs was also found. The overall inconsistency of available information hindered precise assessment of the evidence. There remains much uncertainty as regards the role of prescribing ADs in therapeutic strategies, as well as the appropriateness and accuracy AD prescriptions. It is important to emphasise the difference between assessing the scientifically proven effect of an AD for a certain condition and the potential benefit of an AD at the right dose for a specific patient despite a lower level of evidence. Further studies are required to provide physicians more comprehensive knowledge and to improve patient care.

## Abbreviations

AD(s): Antidepressant(s); ALS: Amyotrophic lateral sclerosis; CDSR: Cochrane database of systematic reviews; Cismef: Catalogue and index of the French-language health resources; HAS: French “haute autorite de sante”; IBS: Irritable bowel syndrome; NICE: English National Institute for clinical excellence; NGC: National guideline clearinghouse; PC: Primary care; SMD: Standardized mean difference; TCA: Tricyclic agent; SSRI: Selective serotonin reuptake inhibitors; SNRIs: Selective norepinephrine reuptake inhibitors.

## Competing interests

The authors declare that they have no competing interests.

## Authors’ contributions

Conception of the idea for the study: AM. Development of the protocol: AM, IAA, JPL, MS, and PB. Data collection and extraction: AM, IAA, JPL. Critical assessment: AM, IAA, JPL, MS, and PB. PVR and LP participated in the design and coordination and helped to draft the manuscript. Writing of the manuscript: AM. All of the authors have read the draft critically, to make contributions, and have approved the final text.

## Pre-publication history

The pre-publication history for this paper can be accessed here:

http://www.biomedcentral.com/1471-2296/14/55/prepub

## Supplementary Material

Additional file 1 Rating of recommendations. Click here for file
