# Supplementary material for: Evidence of prescription of antidepressants for non-psychiatric conditions in primary care: an analysis of guidelines and systematic reviews
Source: BMC Fam Pract. 2013 May 4;14:55. doi: 10.1186/1471-2296-14-55 (PMC3648410; doi:10.1186/1471-2296-14-55)
Supplement: Additional file 1 — Rating of recommendations. [file 1471-2296-14-55-S1.docx]

**Rating of Recommendations**

| **Level A rating** (established as useful/predictive or not useful/predictive) requires at least one convincing class I study or at least two consistent, convincing class II studies. |
| --- |
| **Level B rating** (established as probably useful/predictive or not useful/predictive) requires at least one convincing class II study or overwhelming class III evidence. |
| **Level C rating** (established as possibly useful/predictive or not useful/predictive) requires at least two convincing class III studies. |

| **Class I**: A prospective study in a broad spectrum of persons with the suspected condition, using a "gold standard" for case definition, where the test is applied in a blinded evaluation, and enabling the assessment of appropriate tests of diagnostic accuracy. |
| --- |
| **Class II**: A prospective study of a narrow spectrum of persons with the suspected condition, or a well-designed retrospective study of a broad spectrum of persons with an established condition (by "gold standard") compared to a broad spectrum of controls, where test is applied in a blinded evaluation, and enabling the assessment of appropriate tests of diagnostic accuracy. |
| **Class III**: Evidence provided by a retrospective study where either persons with the established condition or controls are of a narrow spectrum, and where test is applied in a blinded evaluation. |
| **Class IV**: Any design where test is not applied in blinded evaluation OR evidence provided by expert opinion alone or in descriptive case series (without controls). |
